# Lipid oligonucleotides as a new strategy for tackling the antibiotic resistance

**DOI:** 10.1038/s41598-020-58047-x

**Published:** 2020-01-23

**Authors:** Tina Kauss, Corinne Arpin, Léa Bientz, Phouc Vinh Nguyen, Brune Vialet, Sebastien Benizri, Philippe Barthélémy

**Affiliations:** 1 ARNA, INSERM U1212, CNRS 6320, University of Bordeaux, Bordeaux, F-33076 France; 2 MFP, CNRS 5234, Université de Bordeaux, Bordeaux, F-33076 France

**Keywords:** Nucleic acids, Bacterial infection

## Abstract

Antibiotic resistance has become a major issue in public health especially for one of the most used antibiotics; the third-generation cephalosporins. One of the main resistance mechanisms in *Enterobacteriaceae*, is the production of Extended-Spectrum β-lactamases. Here, we demonstrated that the oligonucleotide therapy is an efficient approach to reduce the resistance of bacteria to antibiotic treatment. Lipid oligonucleotides (LONs) were proved to be efficient strategies in both delivering the oligonucleotide sequences in the prokaryotic cells and decreasing the Minimum Inhibitory Concentration of resistant bacteria to a third generation cephalosporin, the ceftriaxone. Accordingly, we demonstrated the strong antimicrobial potential of this LON strategy targeting the ß-lactamase activity on both clinical and laboratory strains. Our results support the concept that the self-delivery of oligonucleotide sequences *via* lipid conjugation may be extended to other antimicrobial drugs, which opens novel ways to struggle against the antibiotic resistance.

## Introduction

Since their discovery, antibiotics have revolutionized the medical treatments of patients with bacterial infections by saving numerous lives^1^. They represent a major therapeutic medical tool, which can be used in many treatments, including infections, chemotherapies, transplantation, and surgery for example. However, antimicrobial resistance (AMR) has been observed at dangerously high levels worldwide^2–7^, alternative therapeutic strategies are urgently needed^8^. Among the different resistance phenomena, the AMR involving the third- generation cephalosporins (3GCs), represent one of the major class of antibiotic used worldwide, has become a major public health issue^9–11^. For this β-lactam family, the main resistance mechanism in enterobacteria, is characterized by the production of Extended-Spectrum β-lactamases (ESBLs). Since 2000s, CTX-M ESBLs have gained prominence and are considered pandemic enzymes^10–12^. The name “CTX-M” refers to their potent hydrolytic activity against cefotaxime (a reference 3GC), but also against ceftriaxone (CFX), another 3GC, with similar activity than CTX, but prolonged elimination half-life. Despite national, european and international strategic action plans to limit antibiotic resistance, the rate of CTX-M-producing enterobacteria continues to increase, especially among the clinical isolates *of Escherichia coli*, which is the main bacterial species encountered in both nosocomial and community infections. Among CTX-M variants, the most widespread type in European countries is the CTX-M-15 enzyme^10–12^. Different approaches have been developed to address AMR, including the improvement of intracellular delivery of the antibiotics^13^, the use of natural lipopeptide antibiotic tripropeptin C^14^, or β-lactamases inibitors^15^, for example^16^. However, in the case of small drug inhibitors for example, inhibitor-resistant β-lactamases (IRTs) have developed over time, indicating that new approaches must be explored^17,18^. Recently, antisense therapy has been identified as potential therapeutic tool for tackling AMR. Oligonucleotides (ONs) hybridize with mRNA, which inhibit the expression of the gene responsible of the resistance (like β-lactamases for instance) *via* different possible mechanisms^19–21^. In this context, ONs represent a promising strategy to restore the resistant bacteria sensitivity to current antibiotics treatments, and in particular 3GCs^20,22^. However, despite their high potential, the cellular uptake of oligonucleotides remains one of the key steps for eliciting their biological activity, as the targeted mRNAs are located inside the cells^21,23^. Recently, we demonstrated that Lipid-oligonucleotide conjugates improve cellular uptake and efficiency of antisense in eukaryotic prostate cancer cells^24^. In the context of AMR, the cellular uptake of ONs inside prokaryotic cells is a critical issue^25^. To overcome this problem, we hypothesize that a lipid modification of ONs (LONs) would enhance their delivery. Herein we describe a novel series of oligonucleotide sequences complementary with those of the most prevalent CTX-M-15 ESBL, featuring a lipid moiety conjugated to the ON extremity to improve their intracellular penetration in prokaryotic cells and a phosphorothioate chemistry (PTO) for enzymatic stability.

## Results

### Synthesis of ON and lipid ON (LON)

**Figure 1:**
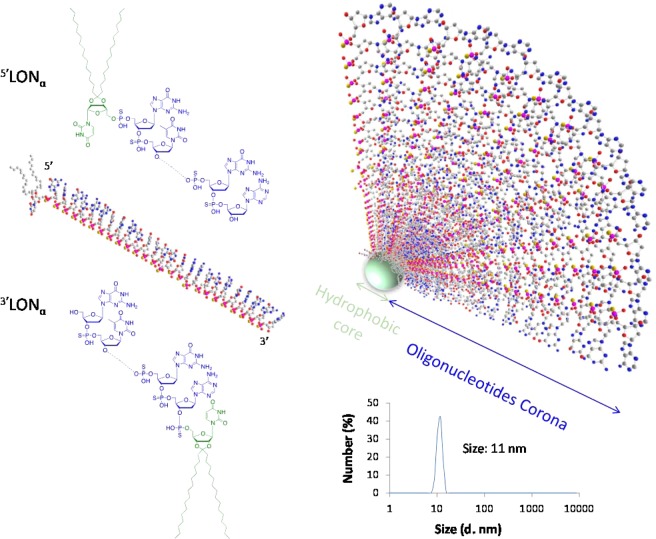
(Left) Chemical structures of lipid antisense (sequence α) conjugates featuring a lipid modification at either 5′ or 3′ extremities, ^5′^LON_α_ and ^3′^LON_α_, respectively. The oligonucleotide sequences (α to δ) used in this study are summarized in Table 1. (Right) Schematic drawing of a micelle involving the ^5′^LON_α_.

All the oligonucleotide based derivatives used in this study were synthesized and characterized as fully described in Supplementary Information. The oligonucleotide ON/LON sequences used were chosen according to literature^20^ and in house developed sequences were synthetized with PTO backbone (Table 1). Briefly, the oligonucleotides were modified at the 5′-end or 3′-end with different lipid phosphoramidites (Fig. 1). The phosphoramidites single chain 1 and 2 were synthesized according to literature procedures and coupled to the 5′-end or 3′ end of the oligonucleotides^26,27^. For each sequence ON and LON were compared. The phosphodiester oligonucleotide (PO) sequences (‘scramble’) were also synthesized as controls wherein the sequence did not target undesired mRNA sequences. All the ONs and LONs were purified by HPLC and later characterized by ESI mass spectrometry (Supplementary Table S2).

**Table 1:** Sequences of tested ONs and LONs.

Name^(a)^	Length (mers)	Sequence (5′ → 3′)
(L)ON_α_^(b)^	25	GCG CAG TGA TTT TTT AAC CAT GGG A
(L)ON_β_	19	CGT GTA GGT ACG GCA GAT C
(L)ON_γ_	25	TGA ACT GGC GCA GTG ATT TTT TAA C
(L)ON_δ_	21	GTC GGC TCG GTA CGG TCG AGA
LON_control_	19	TGT AGT AGG TTG TGT CTG G

^(a)^LONs being 5′ or 3′ conjuguates of ON with ketal bis-C_15_ lipid, ^(b)^Cyanine 5 was conjugated to the 3′ end of 5′(L)ON_α_.

### Physicochemical characterization of micelles

Expectedly, while ONs remain in aqueous solution without specific self-organization (no significant population of objects in water by DLS), LON amphiphiles organize themselves in micelles and larger objects^24,28,29^. The mean size of micellar population of different sequences measured by Dynamic Light Scattering (DLS) in extracellular salt conditions (145 mM Na^+^ and 5 mM K^+^) ranged around 10 nm (Supplementary Table 1), independently on the oligonucleotide sequence, with negative zeta potential (−24 to −37 mV for 5 µM and 30 µM aqueous concentration respectively), as expected regarding polyanion structure of oligonucleotides. The size was shown independent upon LON concentration and room *vs* physiological temperature (Supplementary Fig. 1).

### Bacterial viability

**Figure 2:**
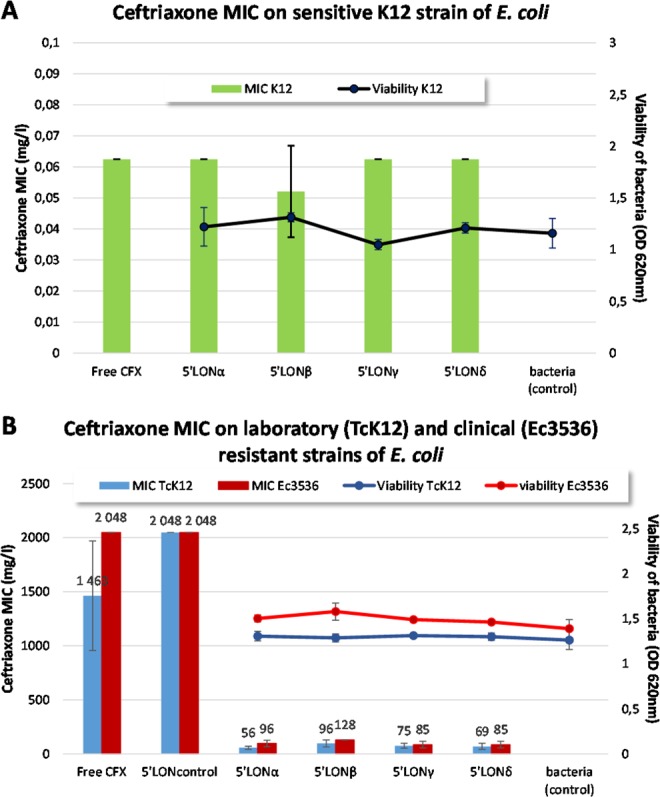
(**A**) Effect of LON sequences, on CFX MIC (left axis) or bacterial viability (right axis) on sensitive K12 strain *E. coli* after 24 h of incubation. (**B**) Effect of LON sequences on CFX MIC (left axis) and bacterial viability (right axis) of resistant laboratory and clinical strain Ec3536.

The effect of ON as well as their lipid conjugates was performed on two *Escherichia coli* laboratory strains: *i.e*. the sensitive strain K12 and its resistant transconjugant TcK12 which contained a conjugative plasmid with the *bla*_CTX-M-15_ gene. The effect of ON/LON was further confirmed on the clinical *E. coli* strain, Ec3536^10^, which also contained *bla*_CTX-M-15_ gene. The results showed that the presence of sequences with lipid conjugates did not affect bacterial viability (Fig. 2A,B right axis, p > 0.48). When tested on sensitive laboratory strain *E. coli* K12, the MIC found in absence of ON sequences was 0.06 mg/L (SD 0, n = 3) of CFX (Fig. 2A left axis). The presence of neither oligonucleotides sequences nor their lipid conjugates affected the MIC significantly (Fig. 2A. left axis).

The effect of ON sequences and lipid conjugates was further tested on the resistant laboratory strain, TcK12. The results (Fig. 2B) show an important decrease of ceftriaxone (CFX) MIC in presence of LONs. Among sequences reported in literature^19^, the corresponding PTO sequence of ^5′^LON_α_ (concentration of 5 µM) was the most potent lipid conjugate for CFX MIC decrease on resistant *E. coli* TcK12 strain, with a 26-fold decrease (means of MICs, 56 mg/L with ^5′^LON_α_ *vs* 1365 mg/L without ^5′^LON_α_, Fig. 2B). As observed on sensitive K12 strain, no effect on MIC nor on bacterial viability was observed (Fig. 2A).

No CFX MIC decrease was obtained with the ^5′^LON_control_, tested in the same conditions (Fig. 2B). These results of CFX MIC decrease were confirmed on the resistant clinical strain of *E coli* Ec3536 (Fig. 2B).

The effect of LONs on MIC was further shown to be dose-dependent (Supplementary Fig. 2). The concentration of 5 µM chosen for the initial screening corresponds to the minimal concentration to reach the minimum MIC.

**Figure 3:**
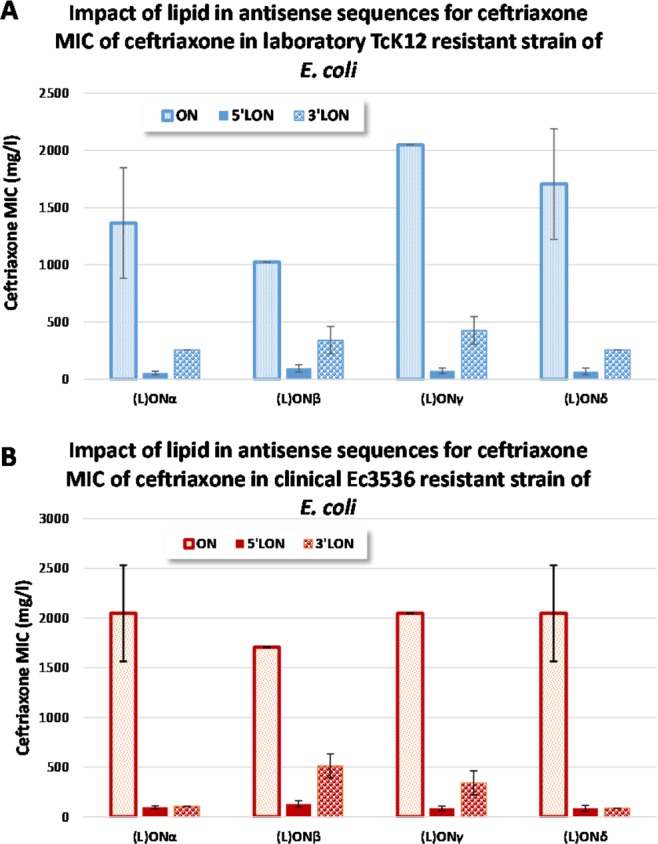
Effect of LON, (modified either at the 5′ or 3′ extremities) on the CFX MIC after 24 h of incubation. Experiments realized on either (**A**) laboratory resistant TcK12 strain or (**B**) clinical resistant Ec3536 strain.

The position of nucleolipid, initially inserted at the 5′ oligonucleotide extremity *via* a 5′-5′ linkage (Fig. 1) was modified to 3′ position. The results showed that while sensitive *E. coli* strains were not affected (Supplementary Fig. 3), resistance of bacteria was partially reversed with 3′ lipid conjugates, but to a lesser extend compared to 5′ lipid conjugates. The result was sequence-dependent and strain dependent (Fig. 3A,B for clinical and laboratory resistant strains respectively).

Beyond the presence of lipid conjugate, the impact of different chemical features was tested on bacterial MIC with control sequences and sequences of interest (Supplementary Table 3). The importance of PTO backbone compared to PO backbone of oligonucleotides was demonstrated (Supplementary Fig. 5A). Furthermore, 19 to 25 pb sequences appeared of appropriate length to provide a decrease of ceftriaxone MIC (Supplementary Fig. 5B).

**Figure 4:**
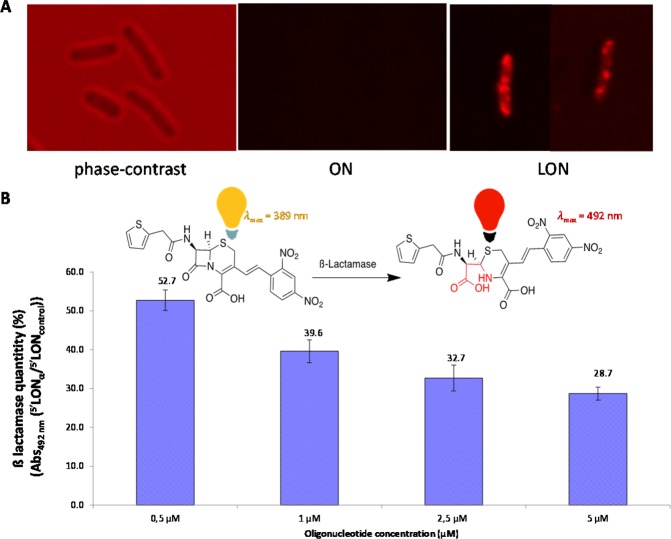
Confocal microscopy imaging of (**A**) *E. coli* TcK12 without laser excitation, (**B**) ß-lactamase quantification in *E. coli* TcK12 in presence of LON_α_ or LON_control_ using a nitrocefin colorimetric dosage.

In order to demonstrate LON intra-bacterial penetration and effect, Cyanine 5 was coupled to the 3′ extremity of ^5^′LON_α_ sequence. While not affecting the MIC (data not shown), the fluorescent microscopy allowed to visualize intra-bacterial localization of ^5′^LON_α_ (Fig. 4A). ON (ON_α_-_Cy5_) resulted only in an enhanced background noise.

The ß-lactamase quantity was investigated by using a chromogenic cephalosporin, the nitrocefin. An inhibition was observed in *E. coli* TcK12 cultivated in presence of different concentrations of LON_α_ compared to LON_control_ (Fig. 4B).

Furthermore, to detect the expression levels of *bla*_CTX-M-15_ mRNA and CTX-M-15 protein in *E. coli* TcK12, relative quantifications were achieved by Reverse Transcription-qPCR and western blotting experiments. Surprisingly, the results could not evidence any significant inhibition of the *bla*_CTX-M-15_ transcription (Student, p > 0.05) (Supplementary Fig. 6A). The preliminary experiment of quantification of CTX-M-15 protein indicated similar levels in presence of ON or LON (Supplementary Fig. 6B) of sequences of interest and control sequences, compared to (L)ON-free untreated control.

## Discussion

In previous studies we explored the ability of hybrid nucleolipids^30^ to form nanoparticles when formulated to different kinds of therapeutic molecules, including small active pharmaceutical ingredients (API)^31^, siRNA^32^, antisenses^33^. It was demonstrated that the resulting nanocarriers displayed higher biological activities compared to the unformulated drugs. We also investigated the impact of the direct lipid conjugation to oligonucleotides^34^ (anti miRNA^35^ or antisenses^24^) on their activity and/or delivery in eukaryotic cells. In all cases, it was found that lipid conjugation was able to improve the delivery and activity in eukaryotic cancer cells. In this study, we generated nucleolipid conjugates featuring oligonucleotide sequences targeting β-lactamase mRNA in resistant bacteria. From our knowledge such a lipid modification has not been investigated in the context of delivering nucleic acids into prokaryotic cells, and especially in Gram-negative bacteria which possess in their bacterial cell wall both peptidoglycan and outer membrane. The aim of this study was to enlarge the self-delivery concept of therapeutic oligonucleotides for antibiotherapy purpose, in order to tackle the antibiotic resistance issue. To validate our approach, a family of oligonucleotide conjugates was investigated with ceftriaxone as a β-lactam antibiotic.

β-lactam, including penicillins, cephalosporins, carbapenems and monobactam are the most used antibiotics for the treatment of bacterial infections^12^. The main targets of these drugs are penicillin-binding proteins (PBPs). It is well documented that the interactions between the β-lactam ring and PBP results in an inhibition of the cell wall’s peptidoglycans synthesis, which induces the bacterial lysis^36,37^. The ceftriaxone (CFX), used in this study, is a broad-spectrum, which belongs to 3GCs. This antibiotic was selected because it is one of the most commonly used antibiotics due to its high antibacterial efficacy, wide spectrum of activity, prolonged half-life allowing once a day dosing and low potential for toxicity^38^. Its widespread use can be explained by its effectiveness in susceptible microorganisms infections of urinary tract, respiratory tract, skin, soft tissue, bone and joint. Also it has been used in infections in immunosuppressed patients, acute bacterial otitis media, genital infections, disseminated Lyme’s disease, bacteremia/septicemia, meningitis, and in surgical prophylaxis of infections^39^. Among the acquired mechanisms of CFX resistance, the production of ESBLs is one of the most common mechanism in enterobacteria. ESBLs’ action mechanism is to cleave the amide bond in the β-lactam ring, resulting in an inactivation of β-lactam antibiotics. In this family, the group of CTX-M β-lactamases and specifically the type CTX-M-15 β-lactamase that are highly resistant to cefotaxime and CFX are the most frequent ESBLs at the worldwide level^11,40,41^. To struggle against this issue, several method of attack have been considered, including the appropriate use of type and dose of antibiotic^42,43^, antibiotics combination and the development of new or chemically modified antibiotics^43–45^. Alternatively, antibiotics carrier systems have been reported to reduce amount and frequency of antibiotic doses^46–50^.

The initial aim of the current study was to propose a new approach based on oligonucleotides and their lipid conjugates, targeting the mRNA sequences coding for the production of CTX-M-15 β-lactamase. In this context, a series of ON featuring phosphorothiate (PTO) chemistry was modified with lipid moiety at either 3′ or 5′ extremities to increase the cellular uptake. Among the synthesized sequences, ON/LON_α_ was taken from literature^20,21^ but synthesized here with PTO (see Table 1) instead of PNA or PMO chemistry. Also, to monitor the localization a Cyanine-5 fluorescent dye was coupled to the 3′ extremity of ON/LON_α-Cy5_. ON/LON_β_ was tested as it was shown not to have any effect on mammalian cells^24^ while presenting a partial alignment with target mRNA. ON/LON_γ_ and ON/LON_δ_ were designed by broadening the specific binding zone between ON/LON_α_ and ON/LON_β_ with *bla*_CTX-M-15_ gene. The previously published sequence of k-LON_SC_^28^ was used as negative control.

As outlined above, cellular uptake is an important feature for the ON strategy. Thus, spherical micellar assemblies with average diameter ranging from 6.5 and 11.6 nm (Table SI1) were observed spontaneously in aqueous media. These micelles would be responsible to the bacteria internalization as observed by confocal microscopy imaging of *E. coli* TcK12 incubated in the presence of LON_α-Cy5_ (Fig. 4). Importantly, as a proof of concept, only the lipid-modified oligonucleotides LON were efficient in decreasing the MIC of ceftriaxone on two different resistant strains (TcK12 and clinical Ec3536), while the corresponding non-lipidic ON did not show any impact on the MIC (Fig. 2B and Supplementary Fig. 4A,B). Also, the LON effect was found to be dose-dependent as revealed by the MIC study achieved on TcK12 at different LON concentrations (Supplementary Fig. 2). A LON concentration of 5 μM was found to be the optimal concentration.

Our initial hypothesis was that the biological activity of LONs was correlated to their affinity for mRNA. Such specific interactions would induce either a RNAse H dependant cleavage or a steric hindrance avoiding mRNA-ribosome interactions, thus leading in both cases to the inhibition of the translation of CTX-M-15 β-lactamase. The inhibition of CTX-M-15 β-lactamase was shown by measuring of its hydrolysis activity on a chromogenic cephalosporin in *E. coli* TcK12 cultivated in presence of LON_α_ compared to LON_control_ (Fig. 4B), supporting a specific inhibition of the β-lactamase by LON sequences compared with the LON control. The decrease of β-lactamase activity was also dependent on the LON concentration.

Furthermore, the results showed that 3′ lipid conjugates were less efficient in reducing the ceftriaxone MIC compared to 5′ lipid derivatives. Several hypotheses could be proposed to explain this behavior, including (i) a more pronounced destabilization of the heteroduplex mRNA-LON in the case of the 3′ lipid-modification, (ii) a steric hindrance avoiding the RNAse H cleavage of the mRNA strand interacting with the 3′ LON extremity, (iii) a less efficient bacteria uptake in the case of 3′ lipid conjugates, or (iv) inhibition of the β-lactamase activity *via* other post transcriptional mechanisms, for example. Noteworthy, the LON_control_ used in this study feature a phosphodiester chemistry and a shorter oligonucleotide sequence, whereas the active LON_α-δ_ are PTO derivatives with 19 to 25 pb. Also, it was found that the non-cytotoxic LONs led to a strong (up till 25-fold in *E. coli* TcK12) MIC decrease. Such an effect using oligonucleotide approach on both clinical and laboratories resistant strains has been never reached before. Next, in order to determine the mechanism of action, RTqPCR and western blotting analysis were achieved. Surprisingly, no significant PTO LONs activities could be demonstrated on the *bla*_CTX-M-15_ gene expression at both mRNA and protein levels, possibly indicating that the decrease of MIC of Ceftriaxone by the LONs might differ from specific antisense mechanisms. Our serendipitous observations in decreasing the antibiotic resistance might be due to either a specific effect of another target, or an off-target mechanism. Nevertheless, our experiments show that the following molecular features are requested to induce a decrease of the Ceftriaxone MIC values: i) PTO chemistry, ii) nucleolipid moieties and a sequence of at least 19 nucleotides. Hence, the fast and remarkable killing of the resistant bacteria strains after LONs treatments would be explained by several possible mechanisms, including i) an important intrabacteria capture of LONs, ii) a decrease of β-lactamase activity thanks to the oligonucleotide sequences interacting directly or indirectly with the β-lactamase enzyme.

In this contribution, we demonstrated the strong potential of the LON strategy in restoring the antimicrobial activities of cephalosphorins against resistant bacteria, with 25-fold decrease of CFX MIC in resistant strains. Our approach, which could be adapted to other antimicrobial drugs or other resistant bacterial species, opens promising perspectives in the struggle against a worldwide public health issue such as the bacterial resistance.

## Methods

### Strains and materials used

*E. coli* strains included the clinical strain Ec3536 collected from a urine sample of the community patient and provided from the MFP Laboratory collection^10^. Its conjugative plasmid containing the *bla*_CTX-M-15_ gene was transferred by conjugation experiment in a laboratory recipient cell of *E. coli* K12. Consequently, the transconjugant *E. coli* TcK12 was resistant to CFX^10^. Mueller-Hinton bacteria culture medium adjusted in calcium and magnesium ions (MH-CA) and microbiology consumable were purchased from Bio-Rad, France.

Ceftriaxone heptahemihydrate di-sodium salt was pourchased from Discovery Fine Chemical (UK), pharmaceutical grade, batch number: 74786.

Methanol and Acetonitrile (HPLC grade) was purchased from VWR (France).

Demineralized water was prepared at the laboratory by ion exchange (Pure Lab Option ELGA) followed by distillation (Water Still Distinction D4000).

### Synthesis, purification and dosage of oligonucleotides (ONs) and Lipid conjugated oligonucleotides (LONs)

The ONs/LONs’ synthesis step was performed on an automated H8 DNA Synthesizer (K&A Laborgeraete, Germany) at the µmolar scale on 1000 Å primer support (loading: 30–100 µmol/g, Link Technologies, Synbase Control Pore Glass). Conventional β-cyanoethyl phosphoramidite chemistry was used. Phosphorothioate linkage was introduced during the synthesis cycle with Sulfurizing Reagent II (3-((N,N-dimethylaminomethylidene)amino)-3H-1,2,4-dithiazole-5-thione from Glen Research).

For LONs, the double-chain nucleolipid (ketal-bis-C_15_-Uridine) was coupled on the synthesizer at the 5′ end or 3′ end of PTO-ONs or PO-ONs.

### Oligonucleotides’ purification

#### Chromatographic analysis of purity and preparative HPLC

All oligonucleotides synthesized were analysed by using High Performance Liquid Chromatography (HPLC) on Elite LaChrom (VWR) system with a Diode detector at 260 nm and injection volume of 20 µl during 15 minutes.

For ONs, hydrophobic column Xbridge oligonucleotide BEH C_18_ (Waters) with particles’ size of 2.5 µm, 130 Å of porosity and 4.6 × 50 mm of geometry was used. The mobile phase with 2.8 ml/min flow used was 70% of 95% of triethylammonium acetate (TEAA) at 100 mM + 5% of Acetonitrile (ACN) at pH 7) and 30% of 20% of TEAA 20 mM and 80% of ACN.

For LONs, Nucleosil C_4_ column with 4 × 250 mm geometry and particles size of 5 µm, 300 Å of porosity (Macherey Nagel) was used with 1.0 ml/min. Analyses were performed with a gradient of 100% A (95% of triethylammonium acetate (TEAA) at 100 mM + 5% of Acetonitrile (ACN) at pH 7) to 100% B (20% of TEAA 20 mM and 80% of ACN) in 10 min before a plateau at 100% B for 2 min. Column returned at 100% A in 2 min for 1 min before the next run.

Oligonucleotides’ purification for LON was performed using preparative HPLC method with column XBridge Protein BEH C_4_ OBD Pre with 30 × 50 mm of geometry, particles size of 5 µM and porosity of 300 Å. The purification was realized with a gradient of 100% A to 100% B in 2 min before a plateau at 100% B for 1 min. Column returned at 100% A for 1 min before the next run. The run use a flow at 56.25 mL/min. The run of analysis was 4 minutes.

#### Desalting method

The columns of Vivaspin Turbo 4 (Sartorius, cut-off 3.5 kDa, membrane Polyethersulfone) were used for oligonucleotides’ desalting. After rinsing, samples were added into the column and centrifuged at 3000 rpm in 30 min. Three washings were made by adding 2 mL of distilled water into the superior part of the tube and then re-centrifuged as previously. 500 µL of distilled water was added on the membrane to re-suspend oligonucleotide and collect it. The membrane was further rinsed 3 times with 500 µL of distilled water.

### ONs/LONs’ assay

The concentration of all ONs and LONs was determined by microvolume spectrophotometer (mySPEC, VWR®) at 260 nm with automatic oligonucleotide detection mode.

### Dynamic light scattering (DLS) characterization

The size of LON’s objects was measured at room temperature using Zetasizer Nano ZS90 (Malvern Instruments Ltd., UK). Size was measured in a specific cell ZEN 0040 (Malvern, France) for NPs and Zeta Potential in a DTS 1070 cell (Malvern, France). Measurement conditions were: material Protein (RI: 1.450; Absorption: 0.001), dispersant water (Viscosity: 0,8872 cP; RI: 1.330) temperature at 25 °C or 37 °C and equilibration time was 120 s. Each test was triplicated.

LONs’ size of micelles was first determined in previously described conditions extracellular salt (145 mM Na^+^ Cl^−^ and 5 mM K^+^ Cl^−^)^28^, first at 30 µM as previously published^24^ and then compared to our 5 µM relevant concentration. Expectedly, LONs presented negative zeta potential, dependent on their concentration, ionic.

### Effect of ONs/LONs on the sensitivity of bacteria to CFX

Determination of MICs of free CFX with or without ONs/LONs was performed on different strains of *E. coli, i.e*. the CFX sensitive strain K12, and the two resistant strains TcK12 and Ec3536.

#### General procedure of MICs determination

MICs of CFX determinations with ONs/LONs were performed by the standard microdilution method in accordance with the standard method of liquid micro-dilution^51^.

The bacterial inoculum was prepared in 0.85% NaCl from overnight colonies on plates at an equivalent to a 0.5 McFarland standard measured with a densitometer (Densimat, BioMerieux). Then the bacterial suspensions were diluted in Mueller-Hinton broth (MH, BioRad) so that the final culture density in the microtiter plates was equal to 5 × 10^4^ CFU in the final volume of 100 µL. The bacterial suspension was afterwards mixed with CFX (serially diluted 2-fold) and ONs or LONs in order to obtain a final volume per well of 100 µL of the microplate. The CFX concentration was adjusted to surround the MIC of each bacterial strain. The concentration of oligonucleotides in well was fixed at 5 µM, except for dose-effect tests. The concentration range of LONs from 0.05 µM to 50 µM was tested. Microplates were incubated at 35 ± 2 °C for 24 h. The MICs was recorded as the lowest concentration where no turbidity was observed in the tubes and they were measured using turbidimeter (Apollo LB 911 (Berthold)) to a wave length of 620 nm. Independent MICs experiments were repeated at least in triplicate.

### Determination of β-lactamase inhibition with colorimetric method

ß-Lactamase activity is measured by hydrolyzing of the nitrocefin, a chromogenic cephalosporin. Nitrocefin degradation lead to a colorimetric product proportional to the enzymatic activity.

An inoculum corresponding to 10^6^ CFU/mL of *E. coli* TcK12 in presence of LON_α_ or LON_control_ was incubated at 37 °C during 5 h. Then, 10 µL of nitrocefin solution (50 mg/L, Thermo Scientific Oxoid^TM^) was added into each well of the microplate and incubated at room temperature for 45 min before reading by using turbidimeter (Apollo LB 911 (Berthold)) at the wavelength of 492 nm. Each measure is realized in triplicate. The blank optical density was subtracted.

### Optical Fluorescence imaging of LONs’ localization in bacterial cells

ON_α-Cy5_/LON_α-Cy5_ at the concentration of 5 µM incubated with 5 × 10^4^ CFU/ml of TcK12 strain during 20 h were observed under confocal fluorescent microscopy with 630x magnification and PMT4 Detector. Laser excitation’s wavelength was 638 nm.

### Relative quantification of bla_CTX-M-15_ mRNA by RT-qPCR analysis

Total RNA was extracted from mid-logarithmic phase cultures of *E. coli* TcK12 in presence of ON/LON (5 µM) by an acid phenol extraction method. mRNA levels were determined by Reverse Transcriptase-PCR experiments using GoTaq qRT-PCR Master kit (Promega) and CFX96 Touch^™^ apparatus (Bio-Rad) according to the manufacturers’ recommendations. The *gapA* gene encoding for a glyceraldehyde-3-phosphate dehydrogenase was included as the reference gene for the normalization analysis. Oligonucleotides designed for RT-PCR experiments were as following: CTXM15_F and CTXM15_R (5′-TGTGCCGCTGTATGCGCAAA-3′ and 5′-TGCTGTGTTAATCAATGCCACAC-3′, respectively) and RT_gap1 and RT_gap2 5′-TGTTGACGTTGTCGCTGA-3′ and 5′-TTTCTGAGTAGCGGTAGTA-3′, respectively).

Relative mRNA levels were expressed according the 2^−ΔΔCt^ method: [(Ct_CTX-M-15_ − Ct_gapA_)_with LON/ON at 5 µM –_ (Ct_CTX-M-15_ − Ct_gapA_)_without LON/ON_]. Our results are expressed as means of 3 experiments (±S.D.) and as a fold-change in comparison with those of (L)ON free, untreated control (without ON/LON), which is considered as 1. The change higher than 1.5 was considered significant.

### Relative quantification of CTX-M-15 by western-blotting analysis

Total proteins were extracted from mid-logarithmic phase cultures of *E. coli* TcK12 in presence of ON/LON (5 µM). After separation on SDS-polyacrylamide gel electrophoresis, proteins were transferred on PolyVinylDieneFluoride membrane (ThermoScientific). CTX-M-15 (33 kDa) production was normalized against the expression of GroEL protein (58 kDa). After primary antibody binding (anti-CTX-M-1 antibody, Cuzabio and anti-GroEL antibody, abcam), and revelation with a peroxidase-conjugated secondary antibody, chemiluminescence signals were obtained by using the Clarity^TM^ Western ECL substrate (Bio-Rad) and the ChemiDoc^TM^ and ChemiDoc MP Imaging System (Bio-Rad). The results were expressed after densitometric normalization (using Image J software) and after comparison with the value obtained for the (L)ON-free untreated control sample. The experimentation was performed twice with reproducible results, and the difference between control and test conditions were considered significant if the fold-change was higher than 1.5.

### Statistical analysis

Student unpaired bilateral t-test was performed for statistical analysis of the results. p < 0.05 was considered as statistically significant.

## Supplementary information

Supplementary information.
