# Supplementary material for: Lipid oligonucleotides as a new strategy for tackling the antibiotic resistance
Source: Sci Rep. 2020 Jan 23;10:1054. doi: 10.1038/s41598-020-58047-x (PMC6978458; doi:10.1038/s41598-020-58047-x)
Supplement: Supplementary file 1 — Supplementary information. [file 41598_2020_58047_MOESM1_ESM.docx]

*Formatted Scientific Reports*

**SUPPLEMETARY INFORMATION**

**Lipid oligonucleotides as a new strategy for tackling the antibiotic resistance**

Tina Kauss*^a,*^*, Corinne Arpin^b,*^, Léa Bientz^b,#^, Phouc Vinh Nguyen*^a,#^*, Brune Vialet*^a^*, Sebastien Benizri*^a^* and Philippe Barthélémy *^a,*^*

*^a^* ARNA, INSERM U1212, CNRS 6320, University of Bordeaux, Bordeaux, F-33076, France,

*^b^* MFP, CNRS 5234, Université de Bordeaux, Bordeaux, F-33076, France.

* corresponding authors

^#^ equivalent contribution

**Supplementary Tables**

Supplementary Table 1 :

| **Name of oligonucleotide sequence** | **Length (bases)** | **Micellar size (nm, DLS number)** |
| --- | --- | --- |
| **^5’^LON_α_** | 25 | 11.6 +/- 0.8 |
| **^5’^LON_β_** | 19 | 10.2 +/- 0.4 |
| **^5’^LON_γ_** | 25 | 10.8 +/- 0.5 |
| **^5’^LON_δ_** | 21 | 6.5 +/- 0.6 |
| **^5’^LON_control_** | 19 | 11.0 +/- 0.2 |

Supplementary Table 1: DLS (number mode) size of micellar population of oligonucleotide assemblies at 30µM, 25°C in extracellular salt conditions (145mM NaCl and 5mM KCl)

Supplementary Table 2 :

| **Name of oligonucleotide sequence** | **M calculated** | **M experimental** | **ΔM** |
| --- | --- | --- | --- |
| ONα | 8091.754 | 8091.756 | -2.76E-07 |
| ONβ | 6153.594 | 6153.584 | 1.60E-06 |
| ONγ | 8066.748 | 8066.741 | 8.39E-07 |
| ONδ | 6819.641 | 6819.618 | 3.39E-06 |
| LONα | 8846.226 | 8847.125 | -1.02E-04 |
| LONβ | 6908.066 | 6908.038 | 4.10E-06 |
| LONγ | 8821.220 | 8821.196 | 2.75E-06 |
| LONδ | 7574.114 | 7574.088 | 3.38E-06 |

Supplementary Table 2. Mass spectrometry data (Calculated and experimental). Mass spectra were recorded in the electrospray ionization (ESI) mode on a Thermo Fisher Q-Exactive. Mass spectra are provided hereinafter.

Supplementary Table 3

| **Name of the sequence** | **Sequence (5’ – 3’)** | **Length (bp)** | **Chemical structure (Backbone +/- lipid)** |
| --- | --- | --- | --- |
| Sequence 1 or 20-mer_1_ | TC TGG TCA CTT ACT TCA CCC | 20 | PTO, PTO-lipid, PO-lipid |
| Sequence 2 | TG GTC ACT TAC TTC ACC CAG | 20 | PTO, PTO-lipid, PO-lipid |
| Sequence 3 | GG TCA CTT ACT TCA CCC AGC | 20 | PTO, PTO-lipid, PO-lipid |
| Sequence 4 | GT CAC TTA CTT CAC CCA GCC | 20 | PTO, PTO-lipid, PO-lipid |
| Sequence 5 | TC ACT TAC TTC ACC CAG CCT | 20 | PTO, PTO-lipid, PO-lipid |
| 7-mer_1_ | ATA TAT A | 7 | PTO, PTO-lipid |
| 7-mer_2_ | GGT ACG G | 7 | PTO, PTO-lipid |
| 13-mer_1_ | TTC CTT ATT CTG G | 13 | PTO, PTO-lipid |
| 13-mer_2_ | GGT TAT TCC TTC T | 13 | PTO, PTO-lipid |
| 19-mer_1_ | CGT GTA GGT ACG GCA GAT C | 19 | PTO, PTO-lipid |
| 19-mer_2_ | TGT AGT AGG TTG TGT CTG G | 19 | PTO, PTO-lipid |
| 20-mer_2_ | CGG CAC ACT TCC TAA CAA CA | 20 | PTO, PTO-lipid |
| 21-mer_1_ | ATA TAT ATA TAT ATA TAT ATA | 21 | PTO, PTO-lipid |
| 21-mer_2_ | CGT GTA GTA CAT GCA GAT C | 21 | PTO, PTO-lipid |
| 25-mer_1_ | ATA TAT ATA TAT ATA TAT ATA TAT A | 25 | PTO, PTO-lipid |
| 25-mer_2_ | TGG ACT AGA CCC TAC TAG GAC TAG A | 25 | PTO, PTO-lipid |

Supplementary Table 3 : Control sequences and sequences of interest used for the study of the impact of chemical features on ceftriaxone MIC

**Supplementary figures**

Supplementary Figure 1: DLS analysis of ^5’^LON_α._ A) DLS size measurement of ^5’^LON_α_ micellar population in different experimental conditions of concentration and temperature (145mM NaCl, 5mM KCl); B) Distribution fit of ^5’^LON_α_ DLS data compared to algorithm

Supplementary Figure 2

Supplementary Figure 2: Dose dependency of 5’LONs’ effect on ceftriaxone MICs in resistant *E. coli* TcK12 stain

Supplementary Figure 3

Supplementary Figure 3: Effect of lipid conjugate (LON) in 3’ or 5’ position compared to non-conjugated (ON) oligonucleotides on the MIC of *E. coli* sensitive K12 strain

Supplementary Figure 4

Supplementary Figure 4: Absence of effect of non-conjugated sequence compared to lipid on ceftriaxone MIC in *E. coli* resistant strains A) TcK12 strain; B) Ec3536 strain

Supplementary Figure 5:

Supplementary Figure 5: Impact of the chemical structure on ceftriaxone MIC in resistant *E. coli* TcK12 of oligonucleotide sequences : (A) impact of PO *vs* PTO backbone and lipid moiety of 20mer oligonucleotides (B) impact of oligonucleotide length for PTO ON and LON

Supplementary Figure 6 :

Supplementary Figure 6: RTqPCR gene expression and Western blot protein quantification in presence of ON/LONα,δ,control in *E. coli* TcK12 expressed as Fold change compared to untreated control: (A) Relative quantification of *bla*_CTX-M-15_ mRNA measured by 2^-ΔΔCt^ method: [(Ct_CTX-M-15_ – Ct_gapA_)_with LON/ON at 5 µM_ – (Ct_CTX-M-15_ – Ct_gapA_)_without LON/ON_]. Relative quantification of *bla*_CTX-M-15_ mRNA results are expressed as means of 3 experiments (± S.D.) and as a fold-change in comparison with those of (L)ON-free untreated control, which is considered as 1. (B) Relative quantification of CTX-M-15 protein levels normalized with GroEL protein expression, was measured by densitometric quantification (Image J software). As for RTqPCR experiments, values were compared as a fold-change with those obtained for the (L)ON-free untreated control sample, considered as 1.
